# Charge Transfer Mechanism in Type II WO_3_/Cu_2_O Heterostructure

**DOI:** 10.3390/nano14242057

**Published:** 2024-12-23

**Authors:** Anna A. Murashkina, Aida V. Rudakova, Tair V. Bakiev, Alexei V. Emeline, Detlef W. Bahnemann

**Affiliations:** Laboratory of Photoactive Nanocomposite Materials, Saint Petersburg State University, 199034 Saint-Petersburg, Russia; a.murashkina@spbu.ru (A.A.M.); aida.rudakova@spbu.ru (A.V.R.); alexei.emeline@spbu.ru (A.V.E.)

**Keywords:** solar energy conversion, photoelectrochemistry, heterostructure, charge transfer, spectral dependence, efficiency, tandem cell, WO_3_, Cu_2_O

## Abstract

In this study, we explore the charge transfer mechanism between WO_3_ and Cu_2_O in heterostructured WO_3_/Cu_2_O electrodes and in a WO_3_||Cu_2_O tandem photoelectrochemical cell. The physical–chemical characterizations of the individual WO_3_ and Cu_2_O electrodes and the heterostructured WO_3_/Cu_2_O electrode by XRD, XPS, and SEM methods confirm the successful formation of the target systems. The results of photoelectrochemical studies infer that in both the heterostructured WO_3_/Cu_2_O electrode and WO_3_||Cu_2_O tandem photoelectrochemical cell, the major mechanism of charge transfer between WO_3_ and Cu_2_O is a realization of the Z-scheme.

## 1. Introduction

**Figure 1:**
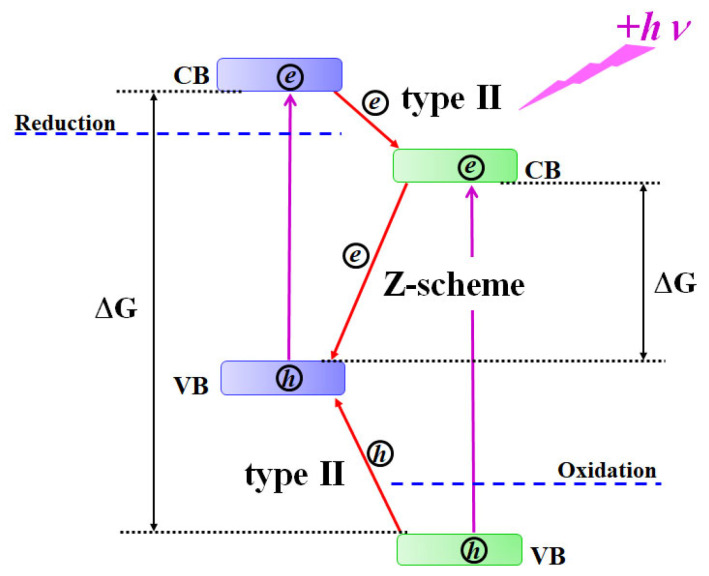
Energy diagram of the electronic band positions and possible charge transfer directions at the heterojunction in type II heterostructures.

In recent years, photoactive materials forming type II heterostructures have been considered as perspective systems for the development of the “green” technology of photochemical solar energy conversion to produce “solar fuel”—either hydrogen from water splitting or higher energy products of carbon dioxide reduction [1,2,3,4,5,6,7]. Type II heterostructures consist of two semiconductor components (SCs), in which energy bands are shifted relative to each other in energy, and the valence band (VB) and the conduction band (CB) of the first SC are located above the corresponding bands of the second SC (see Figure 1).

The major advantage of type II heterostructures consisting of two components compared to conventional single-component photoactive materials is their ability for spatial charge separation between heterostructure components (see Figure 1). This effect results in the decay of charge carrier recombination efficiency and the accumulation of a higher density of both electrons and holes in corresponding components of the heterostructure, which, in turn, increase the probability of multielectron redox reactions, such as water splitting and CO_2_ reduction [7,8]:

2H_2_O + 4e^−^ → H_2_ + 2OH^−^;
2H_2_O + 4h^+^ → O_2_ + 4H^+^;
CO_2_ + 4e^−^ + 4H^+^ → HCHO + H_2_O;
CO_2_ + 6e^−^ + 6H^+^ → CH_3_OH + H_2_O;
CO_2_ + 8e^−^ + 8H^+^ → CH_4_ + 2H_2_O.

In general, there are two different possible mechanisms of charge separation at the heterojunction of the type II heterostructure: the conventional type II mechanism, when electrons transfer from the CB of SC1 to the CB of SC2 and holes migrate from the VB of SC2 to the VB of SC1, and the Z-scheme mechanism, when electrons from the CB of SC2 recombine at the heterojunction with holes in the VB of SC1 (see Figure 1). A distinguished advantage of the Z-scheme compared to the conventional type II mechanism is that the total driving force |∆G| to initiate redox reactions in the Z-scheme is higher than the corresponding driving forces of individual components, whereas for the conventional type II mechanism, the total driving force of the heterostructure is less than the driving forces of individual components (see Figure 1). For example, to split water, the CB of the SC must be located at a potential more negative than the H^+^/H_2_ reduction potential, while the VB must be more positive than the oxidation potential H_2_O/O_2_ (1.23 V relative to the normal hydrogen electrode) [9]. In this context, the extended range of the heterostructure total driving force allows for more redox reactions to be initiated. This makes heterostructures realizing the Z-scheme mechanism more favorable as heterostructured photoactive materials.

To date, numerous studies have been performed to establish charge separation mechanisms in various type II heterostructured photoactive materials, reporting the realization of either conventional or Z-scheme mechanisms. Surprisingly, in different studies, both mechanisms were observed for the same heterostructured materials [7,10,11,12,13,14,15,16,17]. Thus, one can assume that, in general, both mechanisms can be realized in the type II heterostructure at the same time, and which mechanism becomes dominant is dictated by the spatial construction of the heterostructure and photoexcitation conditions as well as by the type of redox processes [7,14,15,16,17,18].

To establish how photoexcitation conditions affect the type of charge separation mechanism at the type II heterojunction, in this study, we explored a heterostructure formed by WO_3_ and Cu_2_O in a photoelectrochemical water-splitting reaction. In addition, we also performed studies of photoelectrochemical behavior in a tandem photoelectrochemical cell with tungsten oxide acting as a photoanode and copper(I) oxide being a photocathode. The selection of these two components of the heterostructure is dictated by their well-established electronic and photoelectrochemical properties [19,20,21,22].

WO_3_ is an *n*-type SC with a band energy of about 2.7–2.9 eV and a sufficiently high oxidation potential of holes, making tungsten oxide a promising visible-light-active material [19,20]. However, the reduction potential of tungsten oxide is insufficient to initiate most of the desired reduction processes. Moreover, the photo-corrosion of WO_3_ during the water-splitting process was evidenced [23,24]. At the same time, a combination of WO_3_ with a complementary semiconductor with high reduction potential might lead to the creation of an efficient redox system in either photocatalytic or photoelectrochemical systems [25].

The role of such complementary semiconductors can be particularly performed by copper(I) oxide, Cu_2_O. Cu_2_O is a narrow band gap *p*-type semiconductor with a band gap of about 2.1 eV [21]. It has a higher reduction potential than that of WO_3_, which makes it an effective photocathode for the photoelectrochemical water-splitting process. However, under irradiation, it can suffer from both self-oxidation, forming copper oxide (CuO), and self-reduction, forming metallic copper (Cu) [21,22].

In spite of the limitations of employing a single-component WO_3_ and Cu_2_O photocatalysts, their combination demonstrated higher activity in both photocatalytic [26,27,28,29] and photoelectrochemical [16,17,30,31,32,33,34] processes. It was shown that *p*-Cu_2_O/*n*-WO_3_ coupling helps to avoid photoinduced charge back reactions in the photoelectrochemical water-splitting process, which results in increased photocatalytic hydrogen production and improved stability of both WO_3_ and Cu_2_O materials [16,17,32,33,34]. The Z-scheme mechanism of charge transfer through the Cu_2_O/WO_3_ heterojunction resulting in the photoinduced water-splitting process was considered in [16,32,33], while in other studies [17,34], it was associated with a conventional type II charge transfer mechanism.

The results of the operation of a tandem photoelectrochemical cell (PEC) with a WO_3_ photoanode and a Cu_2_O|NiO_x_ photocathode confirm the possibility of water splitting without external bias with conversion efficiency achieving about 0.22% under 1 Sun irradiation [35]. The photoelectrochemical behavior of the ITO/WO_3_/Cu_2_O/CuO heterostructured electrode demonstrates an efficiency improved by approximately 5.5 times compared to ITO/WO_3_ due to, as assumed, the conventional type II heterojunction charge separation mechanism [17]. In [36], the charge transfer mechanism in the heterostructure Cu/Cu_2_O/WO_3_ was assumed to coincide with the S-scheme mechanism (a modification of the Z-scheme). Thus, currently, there is no well-established dominating mechanism of charge separation and transfer in the WO_3_/Cu_2_O heterostructure.

In this study, we explore the possibility of charge transfer mechanism variation at heterojunctions depending on the photoexcitation conditions of the planar WO_3_/Cu_2_O heterostructure.

## 2. Materials and Methods

A tungsten (VI) oxide electrode was prepared by the drop-casting of a stable transparent tungsten alcoholate sol. To form the alcoholate sol, tungsten (VI) chloride (≥99.9%, Vekton, Saint-Petersburg, Russia) in an amount of 1 g (2.52 mmol) was dissolved in 20 mL of purified isopropyl alcohol (≥99.0%, Vekton, Saint-Petersburg, Russia) with the addition of 2 mL of glacial acetic acid (60.05 g/mol, Vekton, Saint-Petersburg, Russia). The obtained product was stirred for 30 min, after which 2 mL of concentrated hydrogen peroxide solution (60%, NevaReaktiv, Saint-Petersburg, Russia) was added, and the solution was left stirring for 12 h. After aging, the sol solution was available for synthesis of WO_3_ films for one week.

The films were obtained by uniformly distributing the sol onto a conducting fluorine-doped tin oxide (FTO, 25 mm × 25 mm and a surface resistance of <100 Ohm/cm) substrate surface at room temperature, followed by removing the solvent at 60 °C for 1 h and further annealing the films at 350 °C for 1 h. The heating speed was 60°/h, and the cooling speed was arbitrary. The thickness of the tungsten oxide layer (VI) could be varied by the volume of the applied sol. In operation, the sol volume was 120 μL per 25 mm × 25 mm FTO substrate. The substrates were pre-cleaned by an ultrasonic bath treatment in an aqueous detergent solution, then in isopropyl alcohol, followed by annealing at 450 °C for 30 min.

The Cu_2_O films were prepared by electrodeposition on pre-cleaned conducting FTO substrates from an aqueous solution containing sodium acetate (0.1 M) (99.9%, NevaReaktiv, Saint-Petersburg, Russia) and copper acetate (0.1 M) (99.9%, NevaReaktiv, Saint-Petersburg, Russia). The film deposition process was carried out in a three-electrode electrochemical cell in potentiostatic mode using an Elins-Pro potentiostat (LLC “Elins”, Moscow, Russia) while applying a potential of −0.245 V with respect to the potential of the Ag/AgCl reference electrode (OhmLiberScience, Saint-Petersburg, Russia) for 10 min; a platinum plate (OhmLiberScience, Saint-Petersburg, Russia) was used as the counter electrode. After deposition, the thin layer substrate was washed with distilled water, air dried to remove visible water residues, and annealed in a furnace (LOIP LTD, Saint-Petersburg, Russia) at 300 °C for 5 min, followed by rapid cooling down to room temperature.

Planar “layer-by-layer” heterostructured electrodes were prepared using the methods of synthesis and formation of the corresponding material films based on the preparation methods developed for the individual compounds. The formation of the upper Cu_2_O layer as a component of the heterostructure was also carried out by electrodeposition on a stable WO_3_ film with the same conditions as described above, to form FTO/WO_3_/Cu_2_O heterostructured electrodes. The formation of the heterostructure with an opposite architecture, FTO/Cu_2_O/WO_3_, was not successful since at the heating conditions required to form the WO_3_ layer, the formation of CuWO_4_ takes place.

The surface morphology of the samples was studied using scanning electron microscopy (SEM) with a Zeiss SUPRA 40VP microscope (Carl Zeiss, Oberkochen, Germany). The phase composition of electrodes was determined by X-ray diffraction with a Bruker “D8 DISCOVER” high-resolution diffractometer (Bruker AXS GmbH, Karlsruhe, Germany) applying CuKa radiation in the angle range 20° ≤ 2θ ≤ 80° with a scanning speed of 5.0°/min. The phase reference data were taken from the ICDD database. A Thermo Fisher Scientific Escalab 250Xi spectrometer (Brighton, UK) was used for the registration of both the X-ray Photoelectron Spectroscopy (XPS) and UV Photoelectron Spectroscopy (UPS) spectra.

Electrochemical measurements were performed in a three-electrode electrochemical cell using an Elins-50 Pro potentiostat (LLC “Elins”, Moscow, Russia). The platinum plate and Ag/AgCl electrode were used as the counter electrode and reference electrode, respectively. A 0.2 M aqueous solution of potassium sulfate (pH 6.98, Vekton, Saint-Petersburg, Russia) was used as an electrolyte. The scanning speed for volt–current dependences was 15 mV/s. The Mott–Schottky dependences were registered at 1000 Hz. The spectral dependences of the photocurrent were performed using a home-made two-electrode electrochemical cell with a Pt counter electrode (OhmLiberScience, Saint-Petersburg, Russia).

Photoelectrochemical studies were performed with irradiation by a 300 W Xenon Lamp (Oriel Instruments, Darmstadt, Germany). The light intensity in the visible spectral range applying a cut-off filter JS-11 (Vavilov SOI, Saint-Petersburg, Russia), with a transmittance edge at 410 nm, was 320 mW/cm^2^. The spectral dependences of the photocurrent were measured with monochromator MDR-2 (LOMO, Saint-Petersburg, Russia).

## 3. Results and Discussion

### 3.1. Physical–Chemical Characterization

The surface morphology and side view of the electrodes are shown in Figure S1. The surface of the Cu_2_O electrode consists of agglomerates of microparticles with a size larger than 1 µm, densely adjoining each other. The WO_3_ surface is represented by particles larger than 100 nm, forming a dense layer with a developed surface due to protruding faces of crystallites. The heterostructure FTO/WO_3_/Cu_2_O is characterized by a dense surface morphology, similar to the Cu_2_O phase, and is approximately 0.9 µm in total thickness, composed of individual layers of Cu_2_O with a thickness of 0.4 µm and a WO_3_ layer with a thickness 0.5 µm.

Phase compositions of all the electrodes were characterized by XRD phase analysis. XRD analysis of the electrodes containing Cu_2_O confirmed the presence of the copper(I) oxide phase (card #01-071-3645). It is known that copper(I) compounds can be easily oxidized to copper(II) compounds, and on the conducting substrate, Cu_2_O can recover to metal copper [37,38]. The XRD data shown in Figure S2 demonstrate the absence of both the CuO and Cu phases. The XRD analysis of electrodes based on tungsten oxide revealed the formation of a monoclinic phase WO_3_ with a spatial group P21/N, as presented in Figure S2. For the heterostructured FTO/WO_3_/Cu_2_O electrode, it is shown that the applied methods of heterostructure formation preserve the phase composition of the individual components (Figure S2).

The chemical composition and the oxidation states of the elements were studied by the XPS method. The survey spectra for all electrodes are presented in Figure S3. The main peaks related to the binding energy (BE) of ~933.5 eV, ~38.0 eV and ~529.7 eV correspond to Cu2p, W4f, and O1s, respectively, indicating the expected chemical composition of the studied electrodes.

Figure S4 shows the high-resolution XPS spectra for W4f, O1s, and Cu2p, which were deconvoluted by Gaussians. The Cu2p XPS spectrum of Cu_2_O and the FTO/WO_3_/Cu_2_O sample is characterized as a duplet at 933.4 eV for Cu2p_3/2_ and 953.2 eV for Cu2_p1/2_, corresponding to the Cu^+^ state [39,40,41]. In addition to the characteristic peaks of the Cu(I) state, the peaks at 936.7 eV, 956.5 eV, and a satellite peak at 943.7 eV are observed, indicating the presence of Cu(II) states [39,40,42]. The presence of the Cu(II) state on the surface is rather reasonable because the samples were stored in an ambient oxygen atmosphere, resulting in the partial oxidation of Cu^+^ surface states to more stable Cu^2+^ states [36]. The high-resolution spectra of O1s of the electrodes demonstrate three peaks at ~529.5 eV, ~531 eV, and ~532 eV. According to the NIST (XPS) database for copper oxides [43], the peak at ~531 eV can be attributed to the Cu^+^-O bond, while the peak at ~529.5 eV corresponds to the Cu^2+^–O bond. The peak at the highest energy can be attributed to the surface-bound hydroxyl groups, according to [42].

The WO_3_ peaks at 37.9 and 35.8 eV correspond to the W(VI) state of the electrode material FTO/WO_3_ [44]. The high-resolution spectrum of O1s measured for WO_3_ demonstrates two peaks at 530.5 eV, related to W–O bonds, and 532 eV, assigned to oxygen from the hydroxyl groups [44]. Remarkably, no W4f peak is observed from the heterostructured electrodes due to the dense coating of the inner WO_3_ layer by the outer Cu_2_O layer, indicating the successful formation of a planar layer-by-layer structure. The dense coating of the WO_3_ layer with the Cu_2_O phase is also confirmed by the SEM images (Figure S1).

Figure S5 demonstrates the ultraviolet photoelectron spectra (UPS) of the studied electrodes on an absolute energy scale. The analysis of the UPS spectra provides the energy position of the top of the valence band and the position of the Fermi level by applying the linear intersection method. The corresponding values are presented in Table 1.

Work function (WF) values for all electrodes were measured by the Kelvin probe method and presented in Table 1. Note that the measured WF values are in good accordance with the Fermi level positions determined by the UPS method.

Remarkably, both the Fermi level position and the WF value of the heterostructured electrode are localized between the corresponding values of the individual components of the heterostructure, which indicates a successful formation of the heterojunction.

The transmittance spectra of the planar individual and heterostructured electrodes are shown in Figure S6. The transmittance spectra of WO_3_ and Cu_2_O were transformed in the form of a Tauc plot (Figure S6) to estimate the band gap energies (Eg) of the compounds, whose values are presented in Table 1. As evident from the band gap energy values, E_g_(WO_3_) > E_g_(Cu_2_O), which means that there is a spectral range, 2.4–2.8 eV, where only Cu_2_O absorbs the light in heterostructures.

Note that the application of a Tauc plot for the heterostructured electrode allows for estimating band gap values characteristic for both Cu_2_O and WO_3_, which indicates that the Cu_2_O layer is sufficiently optically transparent to observe the light absorption related to the WO_3_ layer. In other words, both layers in the heterostructure are photoexcited by irradiation in the visible spectral range.

Assuming that the energy of the bottom of the conduction band (E_CB_) can be calculated as:

E_CB_ = E_VB_ + E_g_ (1)

one can estimate the corresponding values presented in Table 1.

**Figure 2:**
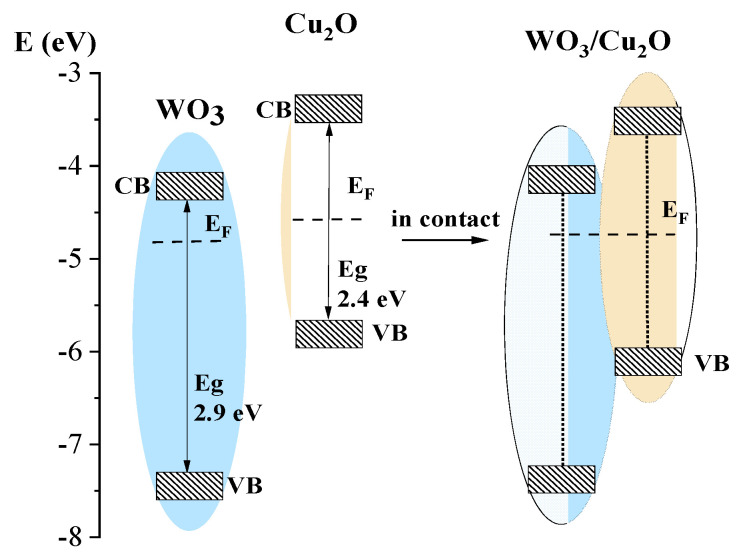
Energy diagram for individual materials and the planar heterostructure WO_3_/Cu_2_O.

Based on the obtained electronic energy characteristics, one can plot the energy diagram for both the WO_3_ and Cu_2_O components and for the heterostructure WO_3_/Cu_2_O (Figure 2). The energy diagrams clearly demonstrate that WO_3_ and Cu_2_O form the type II heterostructure, which potentially can be realized as conventional for type II as Z-scheme mechanisms of charge separation.

### 3.2. Electrochemical Characterization

**Figure 3:**
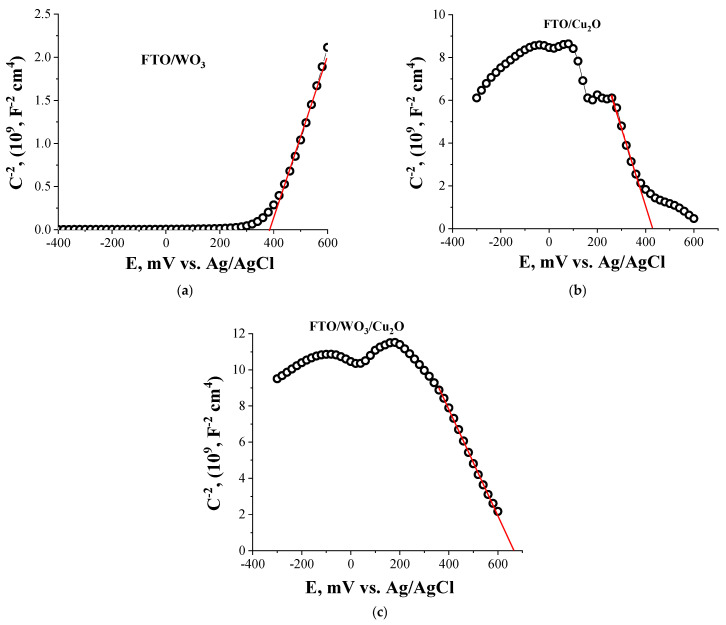
Mott–Schottky plots for FTO/WO_3_ (**a**), FTO/Cu_2_O (**b**) individual component electrodes, and the FTO/WO_3_/Cu_2_O (**c**) heterostructured electrode.

Figure 3 demonstrates Mott–Schottky plots for FTO/WO_3_ (a), FTO/Cu_2_O (b) individual component electrodes, and the FTO/WO_3_/Cu_2_O heterostructured electrode. The positive slope in the Mott–Schottky plot for WO_3_ indicates the n-type conductivity of the SC, while, accordingly, the negative slope observed for the Cu_2_O Mott–Schottky plot corresponds to the p-type conductivity. The flat band potential of the electrodes was evaluated by extrapolating the Mott–Schottky plot slope to the X-axis. The corresponding values of the flat band potentials are 387 mV (vs. Ag/AgCl) for FTO/WO_3_ and 427 mV (vs. Ag/AgCl) for FTO/Cu_2_O electrodes.

The Mott–Schottky plot for the Cu_2_O electrode also demonstrates several characteristic peaks (Figure 3) at specific potential values, which are also observed in the cyclic voltametric (CV) dependences of the electrode (Figure S7a). These reversible characteristic peak CV dependencies can be attributed to the transitions Cu^+^ ↔ Cu^2+^ (~+200 mV vs. Ag/AgCl) and to the formation of Cu^0^ states (~−250 mV vs. Ag/AgCl), which indicates an electrochemical instability of the electrode material. Similar redox transitions were reported in [22]. The CV dependence for WO_3_ is typical for electrochemically stable semiconductors (Figure S5b).

Heterostructured FTO/WO_3_/Cu_2_O electrodes demonstrate p-type conductivity behavior. Therefore, one can infer that heterostructured electrode behavior is dictated mainly by the Cu_2_O outer layer in heterostructures. However, it is wise to note that within the potential range 0–200 mV (vs. Ag/AgCl), a switching in conductivity type from p-type to n-type is observed. This switching can indicate a possible realization of the different regimes of charge transfer through heterojunctions.

Remarkably, the flat band potential value of the heterostructured electrode is shifted toward a more positive potential by 240 mV compared to the flat band potential of the individual Cu_2_O electrode. These shifts correspond to the decrease in the energy positions of the top of the valence bands for the heterostructured electrode compared to the Cu_2_O electrode (see Table 1). Thus, one can conclude that the alteration of the flat band potentials of the heterostructured electrode is induced by the successful formation of a heterojunction between Cu_2_O and WO_3_.

### 3.3. Photoelectrochemical Studies

To clarify the mechanism of charge separation and transfer in the heterostructured FTO/WO_3_/Cu_2_O electrode, we explored the photocurrent behavior (anodic current vs. cathodic current) and spectral dependences of incident photon-to-current conversion efficiencies (IPCEs), which is defined as:

(2) $$IPCE(\%)=\frac{1239.8\cdot j_{ph}}{I_{mchr}\cdot \lambda }\times 100\%$$

where 1239.8 (V nm) is a multiplication of Plank’s constant, h, and the speed of light, c; j_ph_ (mA cm^−2^) is the stationary photocurrent density that was taken from chronoamperometry measurements; I_mchr_ (mW cm^−2^) is the power density of acting monochromatic light; and λ (nm) is the wavelength of this monochromatic light. Also, we explored the photocurrent characteristics in the tandem PEC, where WO_3_ acts as the photoanode and Cu_2_O performs as the photocathode.

**Figure 4:**
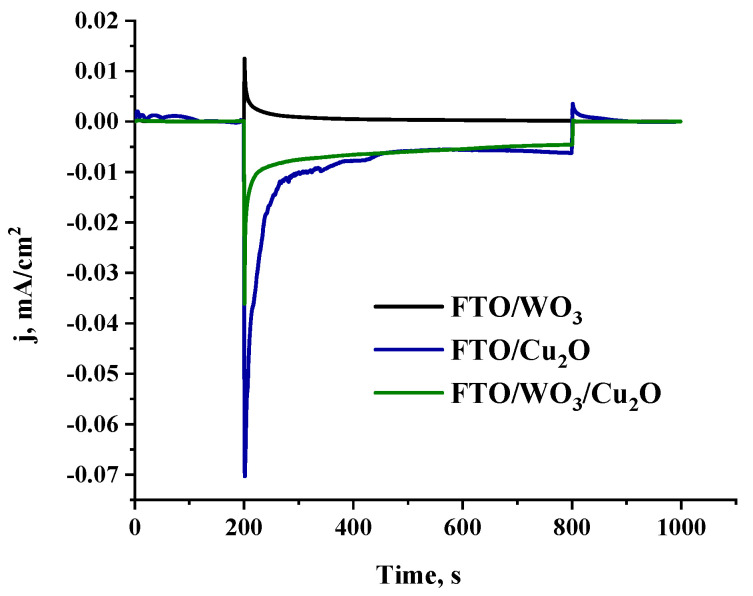
Chronoamperometric dependences of photocurrent density: FTO/WO_3_ (black), FTO/Cu_2_O (navy), and FTO/WO_3_/Cu_2_O (green), without external bias upon irradiation with λ > 410 nm.

Figure 4 demonstrates the chronoamperometric dependences of the photocurrent for the FTO/WO_3_, FTO/Cu_2_O, and FTO/WO_3_/Cu_2_O electrodes under visible light irradiation without external bias.

As expected, the WO_3_ electrode demonstrates typical anodic behavior (positive photocurrent), while the Cu_2_O electrode acts as a typical photocathode (negative photocurrent) under visible light photoexcitation. It is wise to note that the cathodic photocurrent generated by the photoexcitation of the Cu_2_O electrode is significantly larger (about 35 times) than the anodic photocurrent induced by the irradiation of the WO_3_ electrode. Thus, one can conclude that WO_3_ properties could be a limiting factor for photoelectrochemical behavior in both the heterostructured WO_3_/Cu_2_O electrode and the tandem WO_3_||Cu_2_O PEC.

Remarkably, under broad irradiation with visible light, the heterostructured WO_3_/Cu_2_O electrode also demonstrates cathodic behavior typical for Cu_2_O forming the outer layer of the heterostructure. Moreover, the photocurrent generated by the heterostructured electrode is practically the same as the photocurrent generated by the individual Cu_2_O electrode. This infers that charge separation at the heterojunction significantly suppresses the recombination charge carrier losses in WO_3_, which is, apparently, a major factor for the lower activity of the individual WO_3_ electrode.

**Figure 5:**
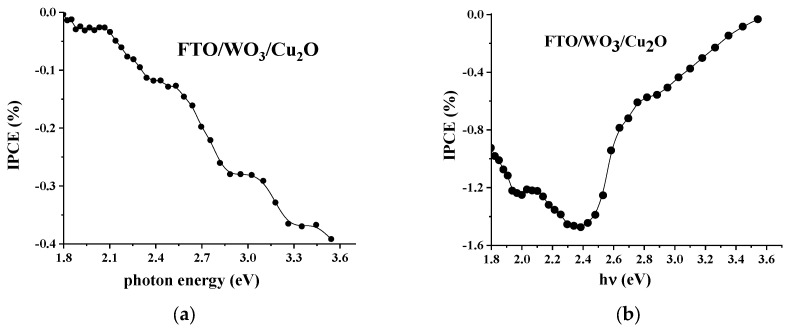
IPCE spectral dependencies of the FTO/WO_3_/Cu_2_O heterostructured electrode under irradiation from the front side (**a**) and back side (**b**).

Figure S8 and Figure 5 demonstrate IPCE spectral dependencies measured for the single-component electrodes and for the heterostructured electrode, respectively.

Spectral dependences of the single-component electrodes (Figure S8), WO_3_ and Cu_2_O, demonstrate typical behavior with maximal efficiency, corresponding to the fundamental absorption of the corresponding photoactive materials, and confirm the anodic and cathodic characteristics for WO_3_ and Cu_2_O, respectively, in the whole spectral range. Remarkably, WO_3_ demonstrates minor activity in the extrinsic absorption spectral region, likely due to the excitation of defect states.

The analysis of the IPCE spectral dependencies obtained for the heterostructured electrode demonstrates several remarkable features of the photoelectrochemical behavior: first, the WO_3_/Cu_2_O heterostructured electrode behaves as a photocathode within the whole spectral range of photoexcitation. Taking into consideration that the spatial structure of the WO_3_/Cu_2_O electrode is planar, this infers that the major pathway of charge separation and transfer at the heterojunction is a realization of the Z-scheme mechanism. The Z-scheme is the only way to observe cathodic photoelectrochemical behavior (cathodic photocurrent) for the WO_3_/Cu_2_O heterostructured electrode since charge separation at the conventional type II heterojunction should result in anodic behavior. Second, unlike Cu_2_O, in which reliable photoactivity starts from the photon energy corresponding to the band gap value (2.4–2.5 eV) and, therefore, reflects the initiation of the band-to-band electronic transitions in Cu_2_O, the photoactivity of the heterostructured electrode is observed at significantly lower photon energies. Specifically, this is brightly demonstrated in the case of the back-side irradiation of the heterostructured electrode (from the WO_3_ layer side). The appearance of the significant photoactivity of the heterostructure within the photon energy range 1.8–2.4 eV can be explained by the photoexcitation of electronic states created at the heterojunction during the formation of the planar heterostructure. In turn, typically, these heterojunction electronic states can play the role of intermediates in heterojunction recombination and, therefore, promote the realization of the Z-scheme of charge separation when both WO_3_ and Cu_2_O components are photoexcited. Third, the observed spectral dependencies of IPCE strongly depend on the irradiation condition: the front side vs. the back side of the electrode. Indeed, front-side irradiation results in an increase in the cathodic efficiency of the heterostructured electrode with an increase in the energy of the actinic photons, while under irradiation from the back side of the electrode, the increase in photon energy leads to a decay in IPCE practically to zero at photon energies corresponding to the fundamental absorption of WO_3_. This behavior correlates with the transmittance spectrum of the heterostructure, where transmittance becomes nearly zero in the same spectral range. This observation indicates that in the case of back-side irradiation, all photons are absorbed within the inner WO_3_ layer of the heterostructure and cannot reach the heterojunction space and the outer Cu_2_O layer.

**Figure 6:**
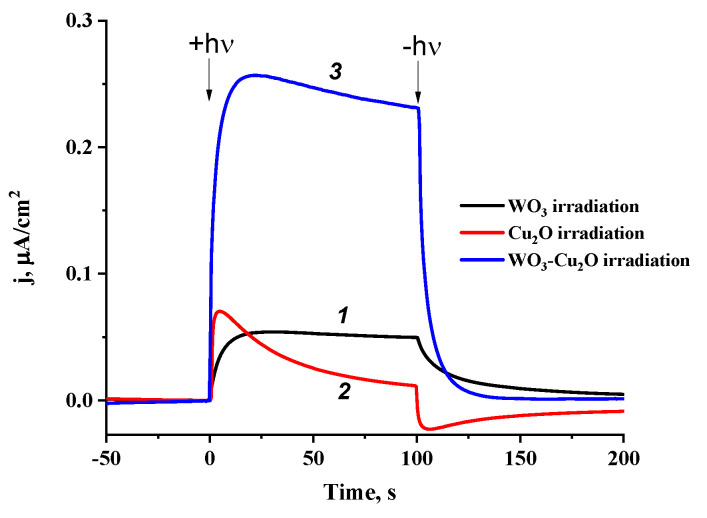
Chronoamperometric dependencies of photocurrent density in the tandem PEC for different irradiation conditions: photoexcitation of the WO_3_ anode only (1), photoexcitation of the Cu_2_O cathode only (2), and photoexcitation of both the WO_3_ and Cu_2_O electrodes (3).

To confirm the realization of the Z-scheme in the WO_3_/Cu_2_O heterostructure, we explored the photoelectrochemical behavior of the tandem PEC with the WO_3_ electrode acting as the photoanode and the Cu_2_O electrode acting as the photocathode. Figure 6 demonstrates the chronoamperometric dependencies of the photocurrent in the tandem PEC for different irradiation conditions: photoexcitation of the anode only, photoexcitation of the cathode only, and photoexcitation of both electrodes.

As evident from the presented data (Figure 6), the photoexcitation of both electrodes results in a significant increase in the photocurrent compared to the photoexcitation of the individual electrodes. Moreover, the effect of photocurrent increase is not additive but rather synergetic.

**Figure 7:**
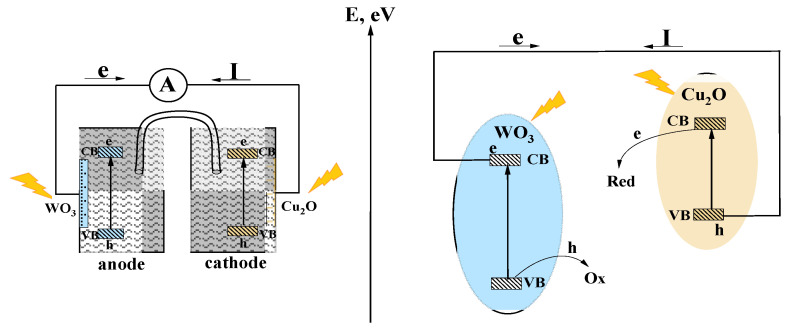
Scheme of electron transfer in the tandem PEC and energy diagram of photogenerated charge transfer in the WO_3_||Cu_2_O tandem system.

According to the energy diagram (Figure 2), the tandem PEC can provide such a synergetic effect and stable anodic photocurrent only because of the realization of the mechanism of charge transfer in the tandem PEC matches the Z-scheme of charge separation and transfer, considering the external circuit as an intermediate interface playing the same role as the heterojunction in the heterostructure (Figure 7).

Thus, based on the presented results, we conclude that the major mechanism of charge separation and transfer in the WO_3_/Cu_2_O heterostructure and in the WO_3_||Cu_2_O tandem PEC is a Z-scheme realization.

## 4. Conclusions

Based on the presented results, we conclude that the major mechanism of charge separation and transfer in the WO_3_/Cu_2_O heterostructure and in the WO_3_||Cu_2_O tandem PEC is a Z-scheme realization. The photoelectrochemical efficiency of the heterostructured electrode also strongly depends on the irradiation conditions, i.e., the wavelength of photoexcitation and the direction of irradiation, either from the front side or from the back side. Irradiation from the back side is strongly blocked by WO_3_ light absorption. Therefore, the realization of the tandem PEC is more favorable from a practical point of view. An essential role in the expansion of the spectral range of the active photoexcitation might be ascribed to the interfacial electronic states at the heterojunction. They also can promote an effective realization of the Z-scheme mechanism.

## Figures and Tables

**Table 1:** Positions of the conduction and valence bands and band gap energies of the heterostructure components with respect to the vacuum energy level.

Electrode	E_g_, eV	E_VB_, eV	E_CB_, eV	E_F_, eV	WF, eV
FTO/WO_3_	2.8	−7.3	−4.5	−4.89	5.0 ± 0.1
FTO/Cu_2_O	2.4	−5.9	−3.5	−4.83	4.8 ± 0.1
FTO/WO_3_/Cu_2_O	2.5 (Cu_2_O) 2.8 (WO_3_)	−6.1	−3.6	−4.85	4.9 ± 0.1

## Data Availability

The main data are provided in this article and the Supplementary Materials. Any other raw/processed data required to reproduce the findings of this study are available from the corresponding author upon request.

## Supplementary Materials

The following supporting information can be downloaded at https://www.mdpi.com/article/10.3390/nano14242057/s1: Figure S1: SEM images of the surface of the studied electrodes; Figure S2: X-ray diffraction patterns of the individual WO_3_ and Cu_2_O and heterostructured WO_3_/Cu_2_O electrodes; Figure S3: XPS survey spectra for the WO_3_ (black line), Cu_2_O (red line), FTO/WO_3_/Cu_2_O (blue line) electrodes; Figure S4: XPS high-resolution spectra of recorded for Cu2p states in FTO/Cu_2_O, FTO/WO_3_/Cu_2_O electrodes, O1S for FTO/WO_3_, FTO/Cu_2_O, FTO/WO_3_/Cu_2_O electrodes, and W 4f for the FTO/WO_3_ electrode; Figure S5: UPS spectra of the WO_3_, Cu_2_O, and FTO/WO_3_/Cu_2_O electrodes (UV monochromatic light source—HeI (21.22 eV)); Figure S6: Transmission spectra and Tauc plots for the Cu_2_O, WO_3_, and heterostructured WO_3_/Cu_2_O electrodes; Figure S7: CV curves of individual and heterostructured electrodes; Figure S8: Spectral dependence of the photocurrent for the WO_3_ and Cu_2_O electrodes.
