# Supplementary material for: Charge Transfer Mechanism in Type II WO3/Cu2O Heterostructure
Source: Nanomaterials (Basel). 2024 Dec 23;14(24):2057. doi: 10.3390/nano14242057 (PMC11678158; doi:10.3390/nano14242057)
Supplement: Supplementary file 1 [file nanomaterials-14-02057-s001.zip › nanomaterials-3361566-supplementary.pdf]

Supporting information

Charge Transfer Mechanism in Type II WO_3/Cu_2O Heterostructure.

Anna A. Murashkina, Aida V. Rudakova, Tair V. Bakiev, Alexei V. Emeline and Detlef W. Bahnemann*

Laboratory of Photoactive Nanocomposite Materials, Saint Petersburg State University, Saint-Petersburg, Russia; a.murashkina@spbu.ru (A.A.M.), aida.rudakova@spbu.ru (A.V.R.), st016840@student.spbu.ru (T.V.B.), alexei.emeline@spbu.ru (A.V.E.)
* Correspondence: detlef.bahnemann@spbu.ru (D.W.B.)

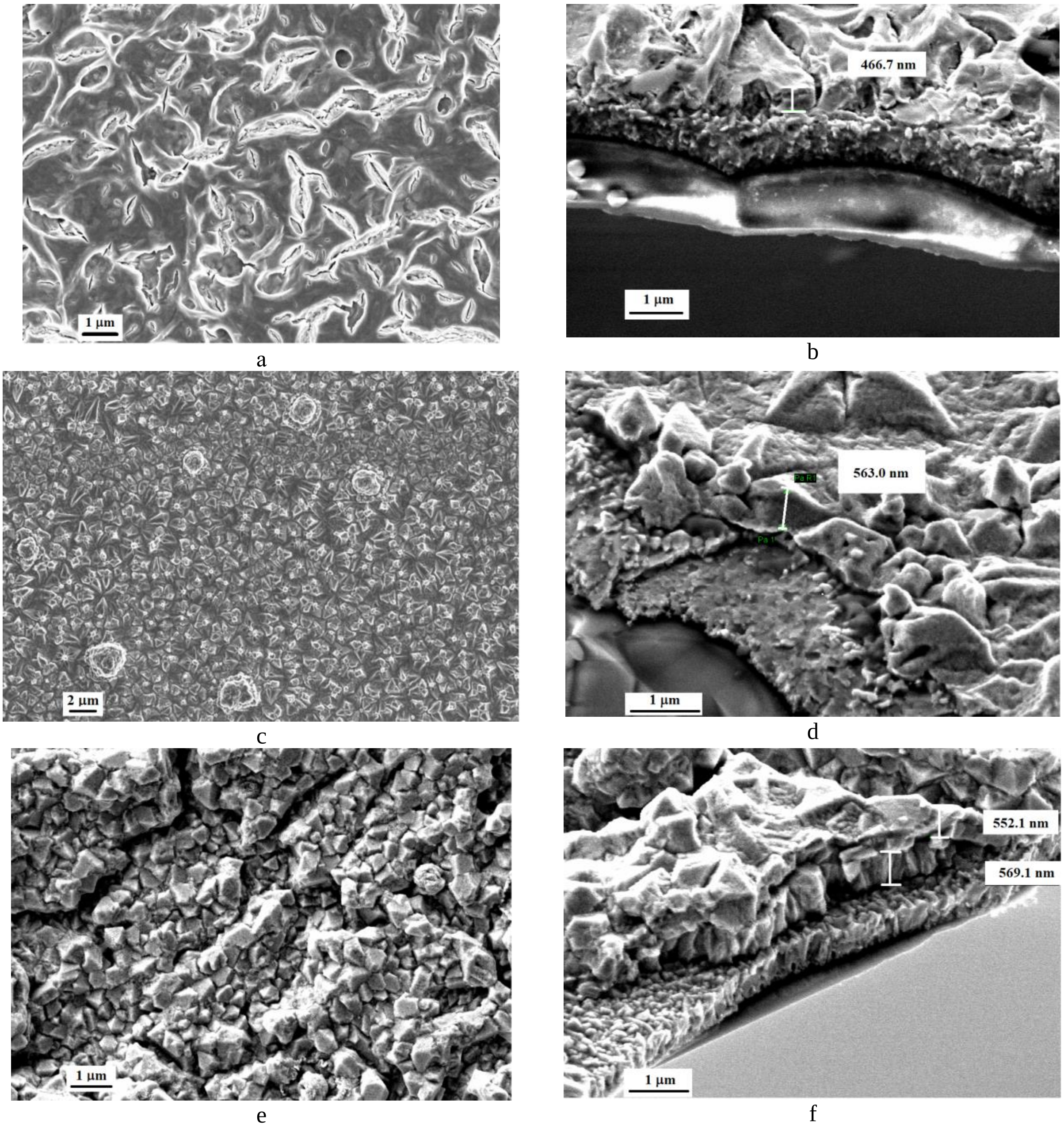

Figure S1. SEM images of the surface of the studied electrodes: FTO/WO_3 (a, b), FTO/Cu_2O (c, d), $FTO/WO_3/Cu_2O$ (e, f), $FTO/WO_3/Cu_2O$ (g, h)

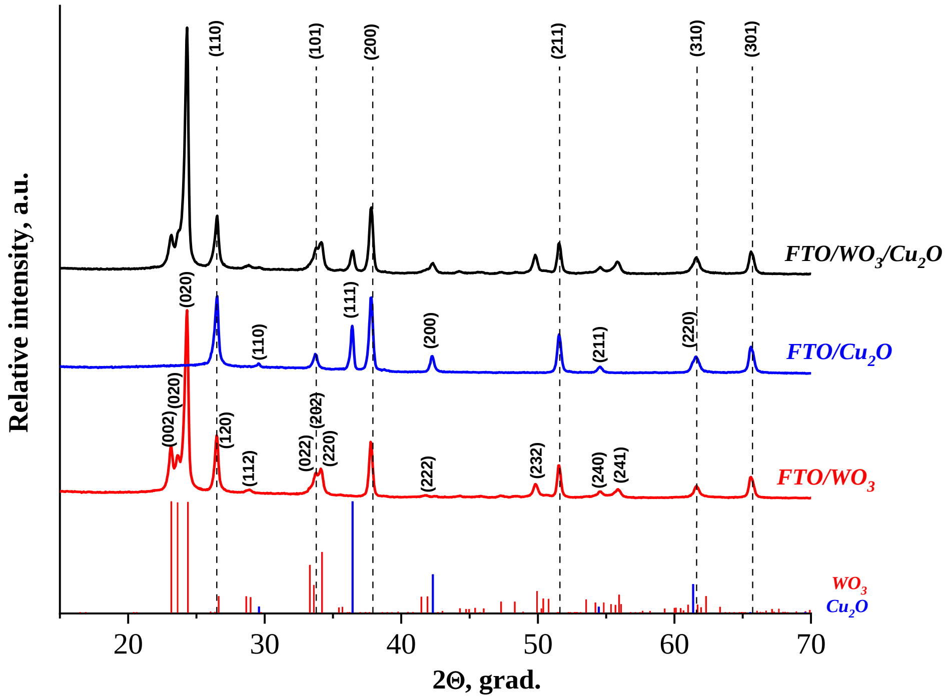

Figure S2. X-ray diffraction patterns of individual WO_3 and Cu_2O and heterostructured WO_3/Cu_2O electrodes. The solid lines mark the major peaks corresponding to the Cu_2O phase (blue, card No. #01-080-7711) and WO_3 phase (red, card No. #01-074-4894) according to the ICDD database. Dashed lines indicate diffraction pattern of SnO_2 phase originated from FTO conductive substrate (Card No. 01-077-0452, ICDD)

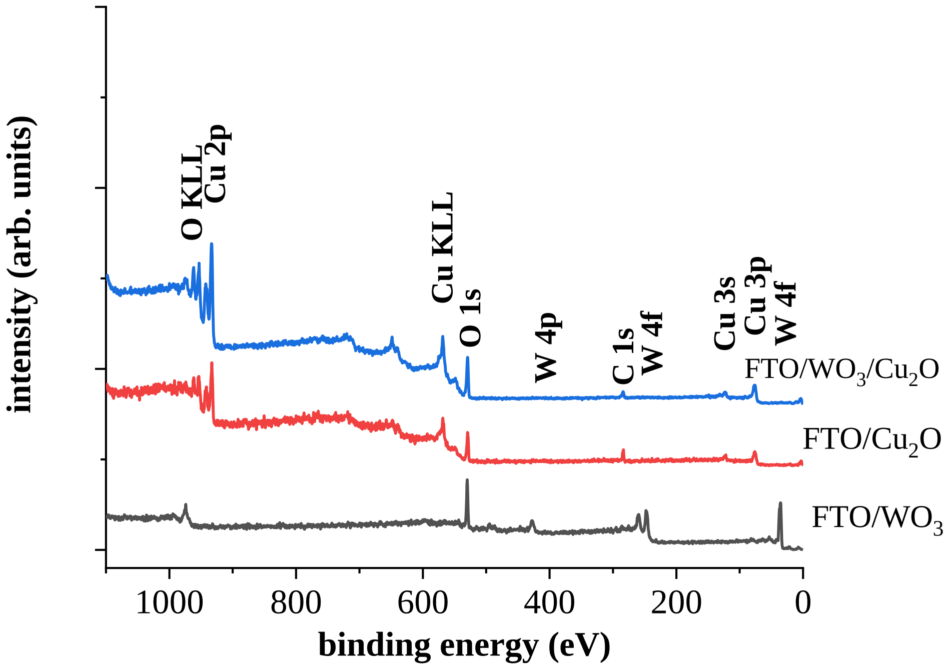

Figure S3. XPS survey spectra for WO_3 (black line), Cu_2O (red line), FTO/WO_3/Cu_2O (blue line) electrodes.

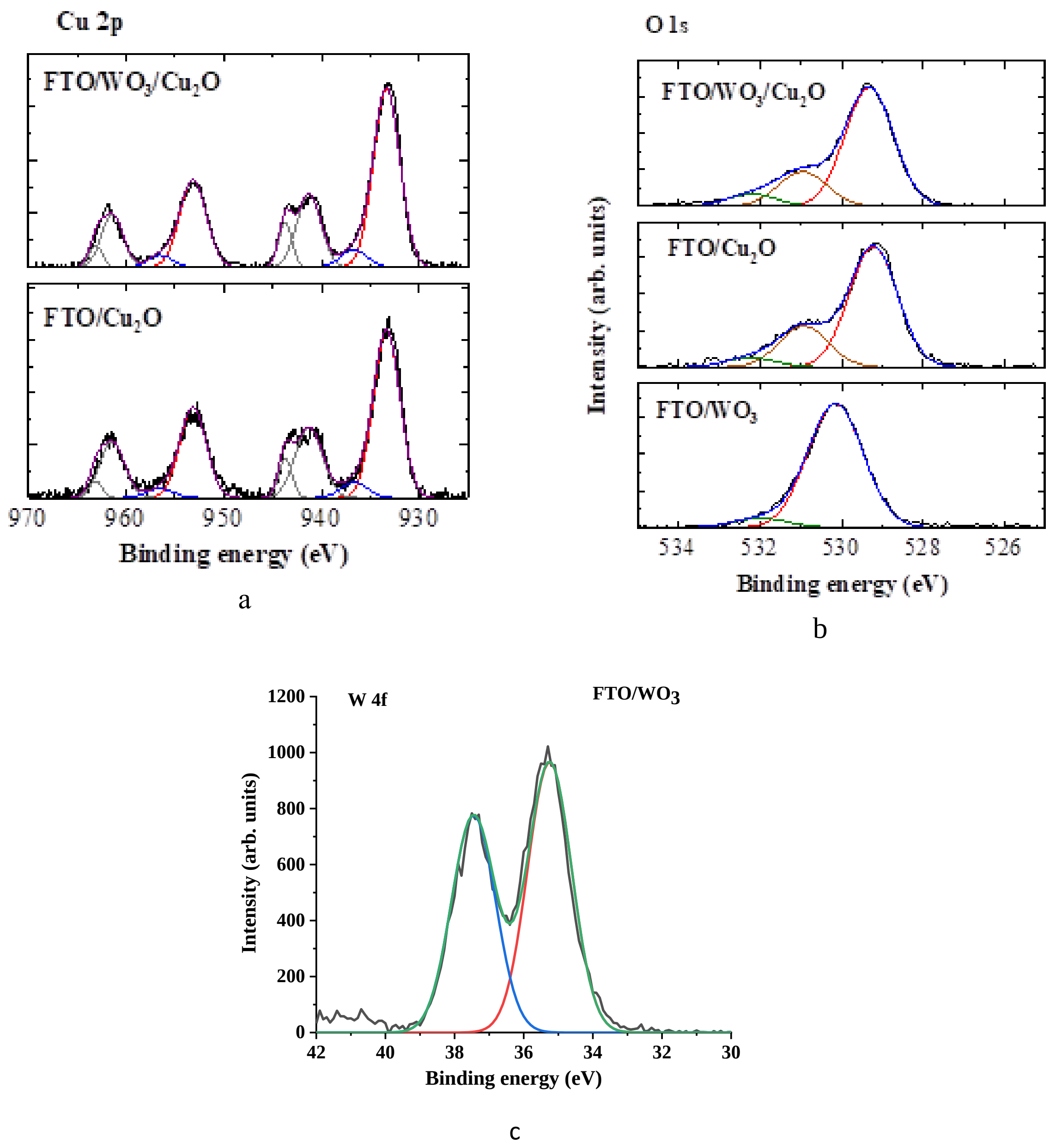

Figure S4. The XPS high-resolution spectra of recorded for (a) Cu2p states in FTO/Cu_2O, FTO/WO_3/Cu_2O electrodes, O1S (b) for FTO/WO_3, FTO/Cu_2O, FTO/WO_3/Cu_2O electrodes, and W 4f (c) for FTO/WO_3 electrode.

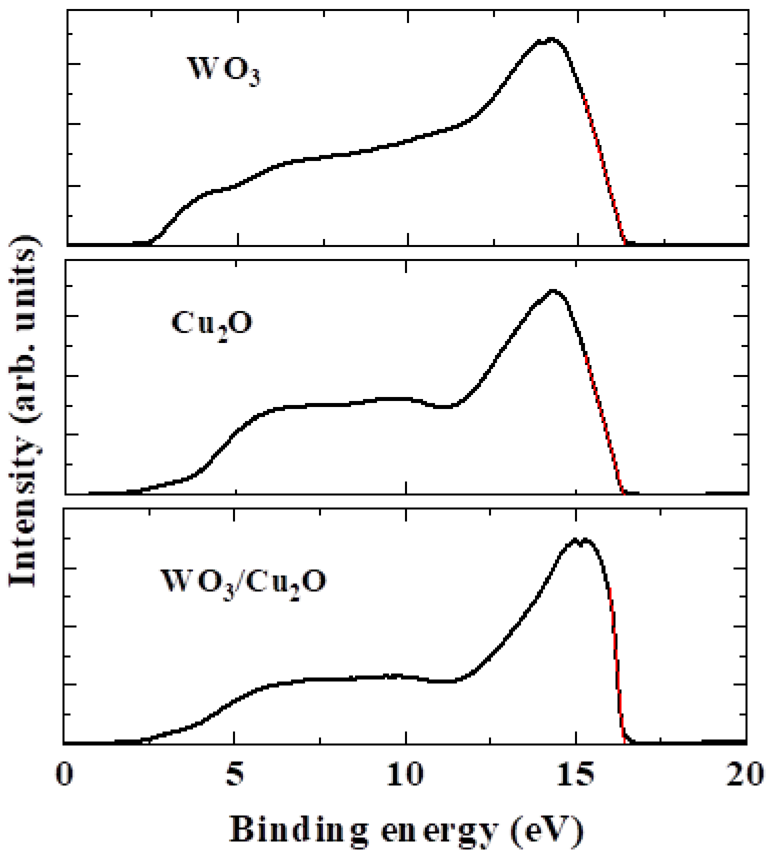

Figure S5. The UPS spectra of WO_3, Cu_2O, FTO/WO_3/Cu_2O electrodes (UV monochromatic light source – HeI (21.22 eV)).

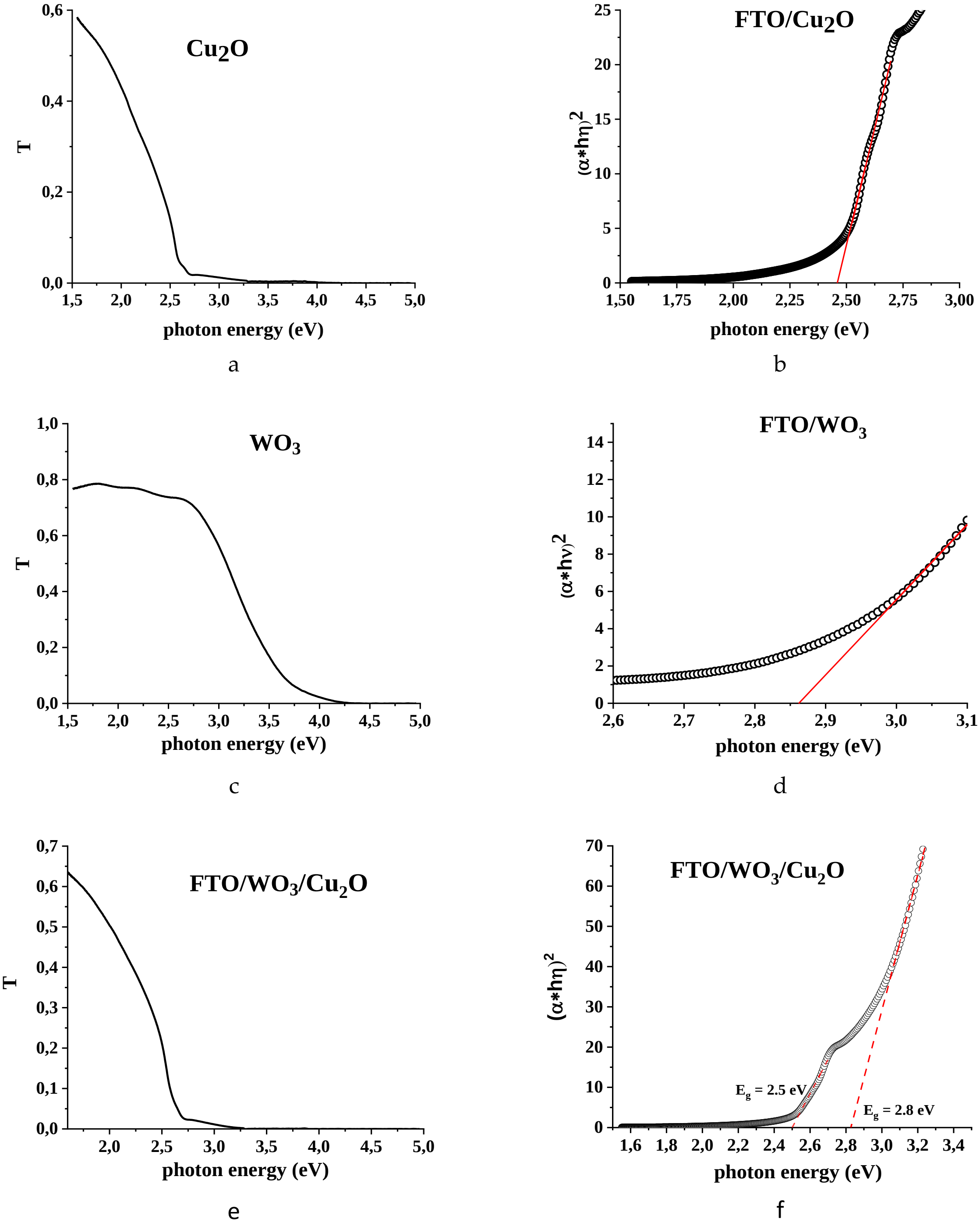

Figure S6. Transmittance spectra T(hν) (a, c, e) and Tauc plots (b, d, f) for Cu_2O (a, b), WO_3 (c, d) and heterostructured WO_3/Cu_2O (e, f) electrodes.

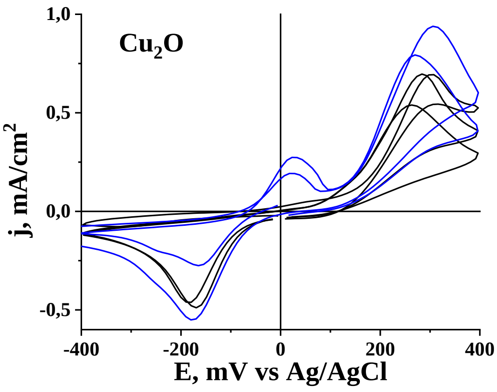

a

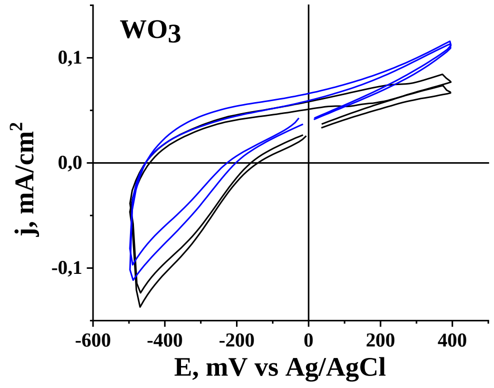

b

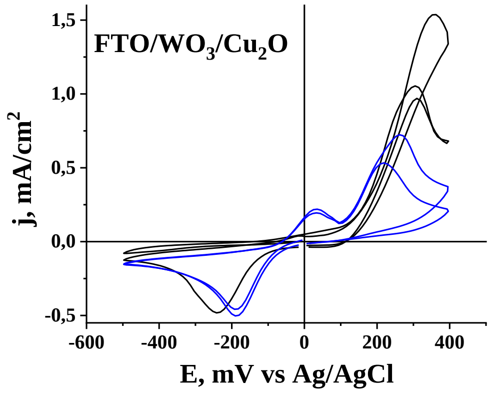

c

Figure S7. CV curves of individual and heterostructured electrodes: FTO/Cu_2O (a), FTO/WO_3 (b), FTO/WO_3/Cu_2O (c) under the dark conditions (black lines) and upon visible light irradiation, $\lambda > 420$ nm (blue lines).

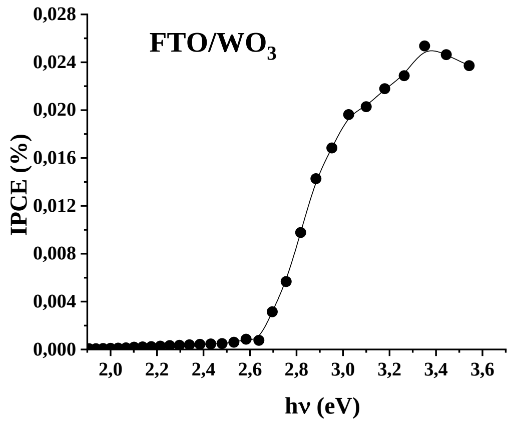

a

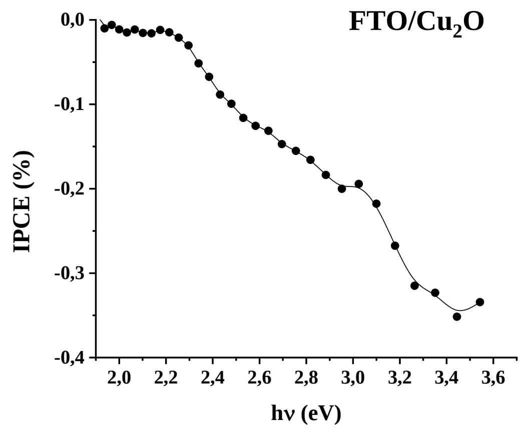

b

Figure S8. Spectral dependence of the photocurrent for WO_3 (a) and Cu_2O (b) electrodes.
